# Modeling Allometric Relationships in Leaves of Young Rapeseed (*Brassica napus* L.) Grown at Different Temperature Treatments

**DOI:** 10.3389/fpls.2017.00313

**Published:** 2017-03-21

**Authors:** Tian Tian, Lingtong Wu, Michael Henke, Basharat Ali, Weijun Zhou, Gerhard Buck-Sorlin

**Affiliations:** ^1^Institute of Crop Science and Zhejiang Key Laboratory of Crop Germplasm, Zhejiang UniversityHangzhou, China; ^2^Department of Ecoinformatics, Biometrics and Forest Growth, Georg-August University of GöttingenGöttingen, Germany; ^3^Institute of Crop Science and Resource Conservation, University of BonnBonn, Germany; ^4^IRHS, INRA, AGROCAMPUS OUEST, University of AngersBeaucouzé, France

**Keywords:** allometry, functional–structural plant model (FSPM), growth function, source–sink relations, temperature, winter oilseed rape, GroIMP

## Abstract

Functional–structural plant modeling (FSPM) is a fast and dynamic method to predict plant growth under varying environmental conditions. Temperature is a primary factor affecting the rate of plant development. In the present study, we used three different temperature treatments (10/14°C, 18/22°C, and 26/30°C) to test the effect of temperature on growth and development of rapeseed (*Brassica napus* L.) seedlings. Plants were sampled at regular intervals (every 3 days) to obtain growth data during the length of the experiment (1 month in total). Total leaf dry mass, leaf area, leaf mass per area (LMA), width-length ratio, and the ratio of petiole length to leaf blade length (PBR), were determined and statistically analyzed, and contributed to a morphometric database. LMA under high temperature was significantly smaller than LMA under medium and low temperature, while leaves at high temperature were significantly broader. An FSPM of rapeseed seedlings featuring a growth function used for leaf extension and biomass accumulation was implemented by combining measurement with literature data. The model delivered new insights into growth and development dynamics of winter oilseed rape seedlings. The present version of the model mainly focuses on the growth of plant leaves. However, future extensions of the model could be used in practice to better predict plant growth in spring and potential cold damage of the crop.

## Introduction

Winter oilseed rape (*Brassica napus* L.) is an important oilseed and fodder crop worldwide (Zhang and Wu, 2007), not only because of its high oil yield (between 40 and 50% of dry mass of seeds), but also because the oil has a high nutritious value, due to the presence of different kinds of aliphatic acids and vitamins (Yang and Tu, 2003). This makes rapeseed oil a very important and healthy ingredient for human nutrition, with a long history as edible oil in Asia and Europe. In addition, if the content of erucic acid is higher than 50%, the seeds can be used as raw ingredient for the production of lubricating oil and suspending agents (Yang and Tu, 2003). Sowing of rapeseed crop mostly starts in September or October at latitudes of 30° in Eastern China. Undergoing vernalization throughout winter, rapeseed blooms in April and harvest in May next year.

A number of climatic and nutritional factors are known to influence both rates and daily integrals of photosynthesis and respiration. Temperature is known to be of utmost importance for photosynthesis and respiration rate (Marcelis, 1996). High temperature leads to a decrease in carboxylation rate as it decreases the specificity of Rubisco for CO_2_ (Lambers et al., 2008), whereas low temperature affects the sucrose metabolism, which leads to accumulation of phosphorylated intermediates and ultimately reduces inorganic phosphates. Finally, inhibition occurs in ATP and photosynthetic accumulation (Chawade, 2011). Besides those effects on photosynthesis, temperature induces some modifications in the morphology of rapeseed. The stems become thinner and the leaves turn thicker under high temperature (Qaderi et al., 2006, 2010). These effects on rapeseed growth (as the decrease of total leaf area) will lead to differences in photosynthesis. Fresh and dry masses have been considered as being positively correlated with temperature during the seedling stage (Rapacz, 1998). Temperature is thus an important factor for photosynthesis rate and consequently growth of rapeseed plants. In order to avoid a potentially considerable reduction in production, it is therefore, important to be able to predict accurately the phenological stage and the topological and geometrical structure of the plant during this period, in order to better assess recovery and survival rates of the crop, which are pivotal for ultimate yield.

To accelerate breeding and optimize the production of rapeseed, the use of crop models as a tool in rapeseed research has been proposed by a number of researchers (Diepenbrock, 2000; Fourcaud et al., 2008; Xu et al., 2011; Robertson and Lilley, 2016). Models for oilseed rape production have been diversified in objectives and methodology. In some classic crop models, the plant canopy was divided into different layers, where the main processes of biomass production were computed for each layer separately and then integrated for the whole crop (Tang et al., 2007, 2009; da Luz et al., 2012). Some models considered the 3D structure and selected functions of the whole plant (Groer et al., 2007). Other models mainly focused on the accurate description of the rates of photosynthesis, respiration, and biomass production at the leaf and whole-plant scale, with local climate parameters as input (Paul and Driscoll, 1997; Müller and Diepenbrock, 2006; Deligios et al., 2013).

In the present study, we investigated the growth and development of young winter oilseed rape plants under different temperature treatments, with a special consideration of the morphology of leaves of different ranks. On the basis of the data established in this study a functional–structural plant model (FSPM) for young rapeseed plants was devised, representing leaf extension kinetics; data about leaf photosynthesis, respiration, biomass production and allocation at the organ and whole-plant scale were obtained from the literature. FSPM being a modeling paradigm that allows not only an instructive visualization of growth and development, but also the integration of heterogeneous datasets in a common scheme, the model we created already helped us to better interpret the correlated datasets of our experiments, apart from the conclusions drawn based on the statistical analyses. However, in this model, we were only able to explore leaf development of young plants. In a further extension of the model, we will eventually use it as a visual decision-support tool for rapeseed breeding and production utilization.

The present model is based on a previous rapeseed model by Groer et al. (2007) and the general sink-source-based FSPM-prototype (FSPM-P; prototype model) by Henke et al. (2016). The FSPM-P is a general model based on the 3D structure of the plant including the physiological characteristics (photosynthesis, maintenance respiration, growth respiration, and plant organ biomass accumulation). The FSPM-P being not designed for a specific crop, it can be adapted in principle to any crop plant with C3 photosynthesis. Its main advantage is that it is easy to parameterize, use, and extend (Henke et al., 2016). In the present study, we discuss how this model could be used to predict the kinetics of storage of carbon reserves (starch) during autumn and winter that might be the key to successful reestablishment of the crop in spring.

## Materials and Methods

### Setup of the Temperature Treatment

Two experiments were set up, differing in planting date. Both experiments focused on young rapeseed plants. Seeds of the leading commercial cultivar of winter oilseed rape (*Brassica napus* L. cv. ZS 758) were germinated in the dark on moist filter paper at 25°C for 2 days. The seedlings were then transferred to plastic pots (height 20 cm, diameter 15 cm) filled with a fertile soil mixture [available N 200 mg kg^-1^, P_2_O_5_ 350 mg kg^-1^, KCl 500 mg kg^-1^, Ca(H_2_PO_4_)_2_⋅H_2_O 3000 mg kg^-1^, K_2_SO_4_ 200 mg kg^-1^, (NH_4_)_2_SO_4_ 500 mg kg^-1^], and cultivated in the greenhouse at a temperature of 10–15°C for 15 days until they had one visible leaf. The potted seedlings were then transferred to a climate chamber (PGX450D, Saifu Instruments, Ningbo, China) and cultivated at three different temperature regimes, combined with a 14-h photoperiod (36 plants per temperature treatment). These temperature regimes correspond to the typical temperature prevalent from September to November at latitudes of 30° in Eastern China^1^. The temperature treatments were: 10/14°C (low temperature, “*L*”), 18/22°C (medium temperature, “*M*”), and 26/30°C (high temperature, “*H*”; night/day temperatures, respectively). Relative humidity was maintained at around 80%. Incident photosynthetically active radiation (PAR) in the temperature chambers was measured above the plant canopy using a Li6400R portable photosynthesis system (LI-COR, Lincoln, OR, USA). It was on average 150 μmol m^-2^ s^-1^, coming from 39 wall-mounted fluorescent light tubes (fluorescent phosphor, type SP41), with a total output of 330 μmol m^-2^ s^-1^.

### Plant Measurements and Analyses

Three plants were chosen randomly every 3 days after putting them into the climate chamber. These plants were photographed, and then sampled destructively for determination of dry weights of each organ. Leaf length, leaf width, petiole length, and leaf area were measured from photographs using ImageJ^®^ 1.41o software (Abramoff et al., 2004). Leaf dry weight was measured after 5 days storage of samples in a drying cabinet at 80°C. Destructive sampling commenced after the plants were put into the temperature chamber and continued for a month. Total leaf number per plant, as well as leaf blade length, leaf blade width, and petiole length were determined for each phytomer (in the sense of Room et al., 1994).

Statistical analyses of total leaf area and dry mass were carried out using the glm procedure of SAS 9.1.3 (SAS^®^ Institute Inc., Cary, NC, USA). The different means of total leaf area and dry mass were based on statistical analyses.

### Leaf Kinetics

**FIGURE 1:**
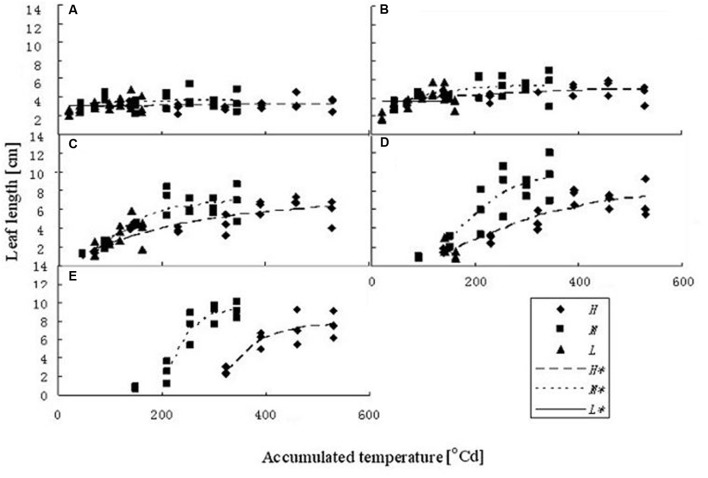
**Leaf length dynamics at three temperature treatments with different leaf ranks.** **(A–E)** Show the length dynamics for different leaf ranks (Ranks 1–5). *H, M*, and *L* represent the data of the leaf length from the experiments under high, medium, and low temperature treatment, respectively. *H^∗^, M^∗^*, and *L^∗^* represent the leaf length growth function according to the leaf length data of the high, medium, and low temperature treatment, respectively.

Leaf kinetics (i.e., extension of the blade in length over time) was determined from the same samples as in Section “Plant Measurements and Analyses” by measuring the lengths of leaf blades every 3 days during their development. The kinetics of a rapeseed leaf blade can be conveniently described using a sigmoid logistic curve, which describes the current length of a blade as a function of plant age (expressed as accumulated temperature). **Figure 1** describes the leaf length development at different temperature treatments, as a function of accumulated temperature, for the first five leaf ranks. To fit the data, a logistic equation was used with three parameters:

$$y_{r,T}=\frac{{y_{m}}_{r,T}}{1+{\left[\frac{ts}{{t_{s0}}_{r,T}}\right]}^{b_{r,T}}}$$

where *y_m_r,T__*[cm] is the maximum leaf length at rank *r* at temperature treatment *T*; *ts*_0_*r,T*__ [°Cd] is the accumulated temperature at which extension rate is maximal; and *b_r,T_* describes the slope of the curve, for a given rank *r* and temperature *T* (**Table 1**).

**Table 1:** Parameters of the logistic curve for three temperature treatments and different leaf ranks in *Brassica napus* seedlings.

Temperature treatment	Parameter	Leaf rank				
		
		1	2	3	4	5
*H*	*ts*_0_	152.66	121.77	246.15	272.08	344.69
	*b*	-0.09	-0.32	-1.10	-2.58	-9.88
	*y_m_*	6.08	7.99	9.04	8.73	7.74
*M*	*ts*_0_	152.91	41.74	117.05	192.93	228.26
	*b*	-0.11	-1.05	-2.48	-4.05	-11.67
	*y_m_*	6.95	5.94	7.36	10.25	9.27
*L*	*ts*_0_	215.52	167.12	12.16	157.92	–
	*b*	48.29	62.99	-4.02	11.01	–
	*y_m_*	3.08	3.69	4.46	2.62	–


**H, M*, and *L*: high, medium, and low temperature treatments.*

### FSPM of Rapeseed Development

The dynamic FSPM of young rapeseed was developed by modification and reparameterization of the FSPM-P (Henke et al., 2016), a fully functional prototype model written in the language XL (Kniemeyer, 2008), and running on the Growth Grammar-related Interactive Modeling Platform (GroIMP) modeling platform (Kniemeyer et al., 2007). Additionally, elements were used from a previous model of rapeseed development by Groer et al. (2007). Essentially, the present model comprised modules for the main biophysical and physiological processes observable in crop plants: light interception, photosynthesis, assimilate partitioning, growth, and respiration.

Light interception was modeled using the twilight light model provided within the GroIMP platform: This model is based on a Monte Carlo raytracer and allows the computation of the spatial and temporal light distribution for suitable objects (such as leaves) in a simulated scene [for details see Buck-Sorlin et al. (2011)]. The climate chamber used in reality contained 39 wall-mounted light tubes (fluorescent phosphor, type SP41). As this setup would have been too expensive in computational terms, it was reconstructed in GroIMP using area lights associated with parallelogram objects representing the two lateral walls and the back wall, with a total output of 330 μmol m^-2^ s^-1^. Two virtual light sensors were placed inside the chamber, above each one of the two shelves. Simulations reproduced the measured photosynthetic photon flux density (PPFD), 150 μmol m^-2^ s^-1^. Virtual lamps, sensors, and the construction elements were specified as geometric objects with optical properties, as described in Buck-Sorlin et al. (2009).

To model leaf photosynthetic rate the model LEAFC3 was used (Nikolov et al., 1995). LEAFC3 is a model of the short-term steady-state fluxes of CO_2_, water vapor, and heat from leaves of C3 plant species, explicitly coupling all major processes involved in photosynthesis (biochemistry of assimilation process, stomatal conductance, and leaf energy balance). The model was parameterized using rapeseed-specific parameters derived from the literature (Liu et al., 2003; Ma et al., 2009).

### Dry Matter Dynamics at Plant Level

To model the temporal dynamics of the integral of dry matter accumulation as a function of accumulated temperature at the plant level, a modified logistic function was used (Li and Ma, 2012) as proposed by Wang (1986). Furthermore, in order to improve the comparison of the dynamics at different temperatures, the input data were normalized, yielding the following equation:

$$y^{\prime }=\frac{y_{m}}{1+e^{{ats}^{\prime }2+{bts}^{\prime }+c}}$$

where *y′* is the normalized dry mass (*y/y_max_*), *ts′* is the normalized accumulated temperature (*ts/ts_max_*), *y_m_*, *a*, *b*, and *c* are shape parameters (**Table 2**). Inspection of the curves of the normalized data yielded that for some leaf ranks and temperature treatments a double logistic function with a transition point at an accumulated temperature *ts_tr_* would be more suitable:

$$y^{\prime }=\left\{ \begin{array}{c} {ts}^{\prime }<{{ts}^{\prime }}_{tr}:\frac{{y^{\prime }}_{m1}}{1+e^{a}1^{{{ts}^{\prime }}^{2}+b_{1}{ts}^{\prime }+c_{1}}}\\ {ts}^{\prime }\geq {{ts}^{\prime }}_{tr}:\frac{1}{1+e^{a}h^{{{ts}^{\prime }}^{2}+b_{h}{ts}^{\prime }+c_{h}}} \end{array}\right.$$

**Table 2:** Parameters of the two growth functions fitted to the normalized time series data of total dry matter in *B. napus* seedlings grown at three temperature treatments.

Parameter	Temperature treatment		
	
	*H*	*M*	*L*
*ym_l_*	7.58	1.10	7.57
*a_l_*	23.99	0.05	7.90
*b_l_*	-16.06	-5.56	-8.23
*c_l_*	6.15	3.72	4.70
*ts_tr_*	0.38	0.76	0.53
*ym_h_*	1.00	1.00	1.00
*a_h_*	-20.82	-106.04	-2.37
*b_h_*	16.49	175.29	-3.99
*c_h_*	-2.07	-73.18	2.88


**H, M*, and *L*: high, medium, and low temperature treatments.*

where *a*_1_, *b*_1_, *c*_1_, *y*_*m*1_, *a_h_*, *b_h_*, *c_h_*, and *y_mh_* are the shape parameters (**Table 3**) for the two logistic curves below and above the threshold accumulated temperature *ts′_tr_*, respectively.

**Table 3:** Parameters of the growth function fitted to the normalized time series data of dry matter in *B. napus* with different leaf ranks at three temperature treatments.

Temperature treatment	Leaf rank						
	
	Parameter	1	2	3	4	5	6
*H*	*ym_t_*	0.78	0.93	0.32	16.91	0.96	16.35
	*a_t_*	45.49	18.54	-6.63	9.16	-16.11	5.46
	*b_t_*	-48.05	-22.77	-29.28	-16.26	4.44	-14.69
	*c_t_*	2.79	2.72	5.66	10.04	4.82	11.96
	*ts_tr_*	–	–	0.61	–	–	–
	*ym_h_*	–	–	15.31	–	–	–
	*a_h_*	–	–	8.20	–	–	–
	*b_h_*	–	–	-16.29	–	–	–
	*c_h_*	–	–	10.63	–	–	–
*M*	*ym_t_*	0.86	0.92	0.83	0.95	1.02	14.07
	*a_t_*	25.33	0.21	-21.35	7.03	17.91	15.86
	*b_t_*	-29.04	-7.69	1.69	-17.70	-40.13	-35.45
	*c_t_*	2.23	1.55	2.90	8.10	19.44	22.16
*L*	*ym_t_*	0.90	6.83	38.46	–	–	–
	*a_t_*	11.62	4.14	11.42	–	–	–
	*b_t_*	-14.74	-5.85	-19.47	–	–	–
	*c_t_*	1.82	3.98	12.18	–	–	–


**H, M*, and *L*: high, medium, and low temperature treatments.*

In this way, the growth function for each leaf rank under different temperature treatments was obtained. With the exception of the third leaf at the high temperature treatment, which required the double growth function (Equation 3) the expansion kinetics of all leaves could be expressed using Equation 2.

As the plants were grown under conditions of potential production (ample water and nutrients, free of weeds, pests and competition), it was assumed that the measured growth corresponded to the potential growth at the given temperature.

### Leaf Area Dynamics at the Whole-Plant Level

The dynamics of leaf area at the level of the plant as a function of the accumulated temperature was modeled using a logistic curve, with an exponential, linear and maturation phase of growth, an equation with four parameters (i.e., twelve parameters in total, as the data were fitted for each temperature treatment):

$${LA}_{t}={LA}_{0,t}+\frac{{LA}_{\max,t}}{1+{\left({ts}_{t}-{TS}_{m,t}\right)}^{-b}}$$

Where *LA*_0_*_,t_* is the initial leaf area; *LA_max_,_,t_* is the maximum leaf area at a given temperature treatment; *ts_t_* is the accumulated temperature at a given temperature treatment; *TS_m,t_* is the accumulated temperature at which extension rate is maximum at a given temperature treatment and *b* describes the slope of the curve (**Table 4**).

**Table 4:** Parameters of the leaf area function in *B. napus* seedlings under three temperature treatments.

Parameter	Temperature treatment		
	
	*H*	*M*	*L*
*LA*_0_*_,t_*	17	10	5
*TS_m,t_*	380	270	135
*LA*_max_*_,t_*	119	184	33
*b*	0.020	0.019	0.025


**H, M*, and *L*: high, medium, and low temperature treatments.*

### Framework of the Rapeseed Model

**FIGURE 2:**
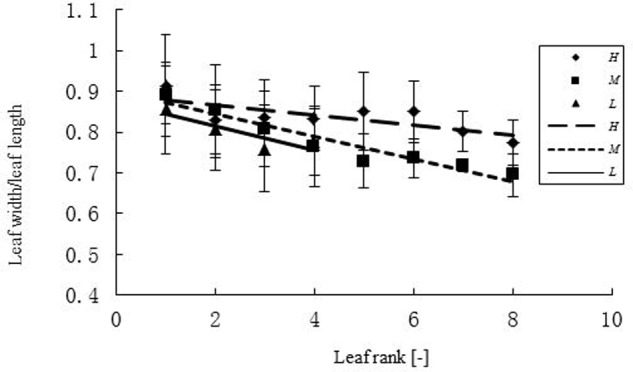
**Width-length ratio (WLR) of leaves at different ranks under three temperature treatments.** *H, M*, and *L* represent the plants under high, medium, and low temperature treatment, respectively. The regression equation for the high temperature was: WLR = -0.0126r + 0.08921 (*R*^2^ = 0.5674, *n* = 108); for the medium temperature: WLR = -0.0276r + 0.8988 (*R*^2^ = 0.9233, *n* = 111); for the low temperature: WLR = -0.0296r + 0.8738 (*R*^2^ = 0.77, *n* = 63).

**FIGURE 3:**
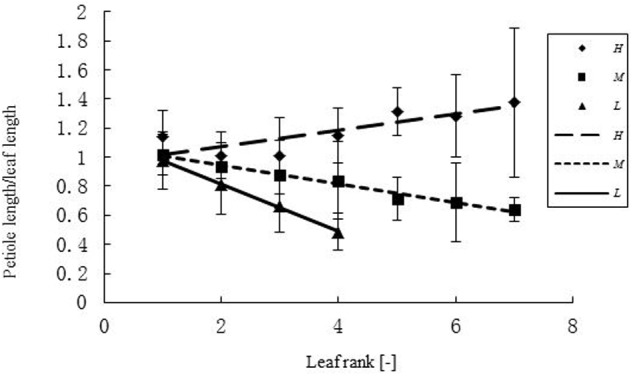
**Differences in petiole length – leaf blade length ratio (PBR) of leaves at different ranks under three temperature treatments.** *H, M*, and *L* represent the plants under high, medium, and low temperature treatment, respectively. The ratio of petiole length and leaf blade length (PBR) differed between temperature treatments for different ranks *r*. For the high temperature it was: PBR = 0.056*r* + 0.9591 (*R*^2^ = 0.6925, *n* = 108); for the medium temperature: PBR = -0.0636*r* + 1.0704 (*R*^2^ = 0.979, *n* = 111); for the low temperature: PBR = -0.1609*r* + 1.1353 (*R*^2^ = 0.9985, *n* = 63).

**FIGURE 4:**
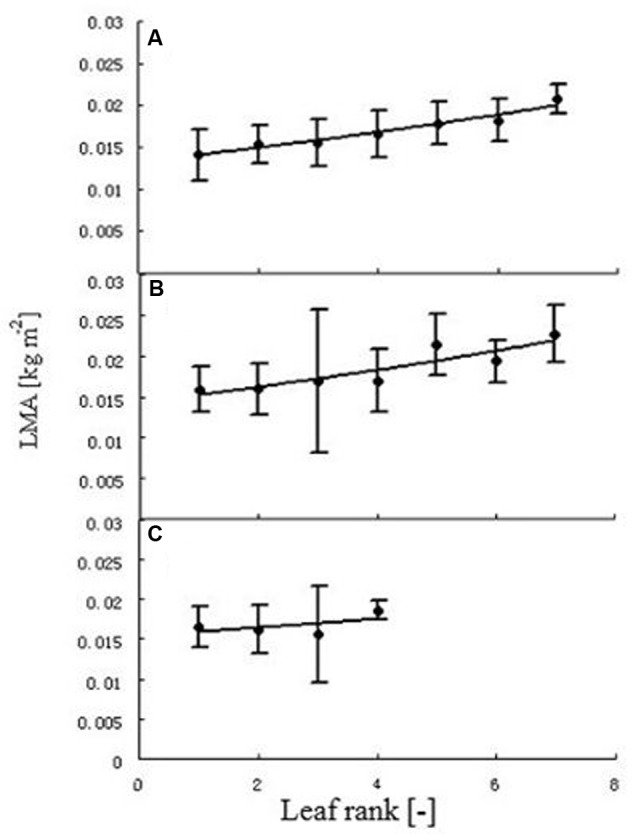
**Leaf mass per area (LMA) at different leaf ranks.** Each point represents the mean of all individual leaves with the same rank. Error bars are standard deviation. Plants under high **(A)**, medium **(B)**, and low **(C)** temperature treatment. The regression between leaf rank (*r*) and LMA was, for high temperature: *LMA* = 0.0133*e^0.0585r^* (*R*^2^ = 0.9585, *n* = 102); for medium temperature: *LMA* = 0.0143*e^0.0607r^* (*R*^2^ = 0.8195, *n* = 108). As the relationship at low temperature was not significant, no regression equation is provided.

**FIGURE 5:**
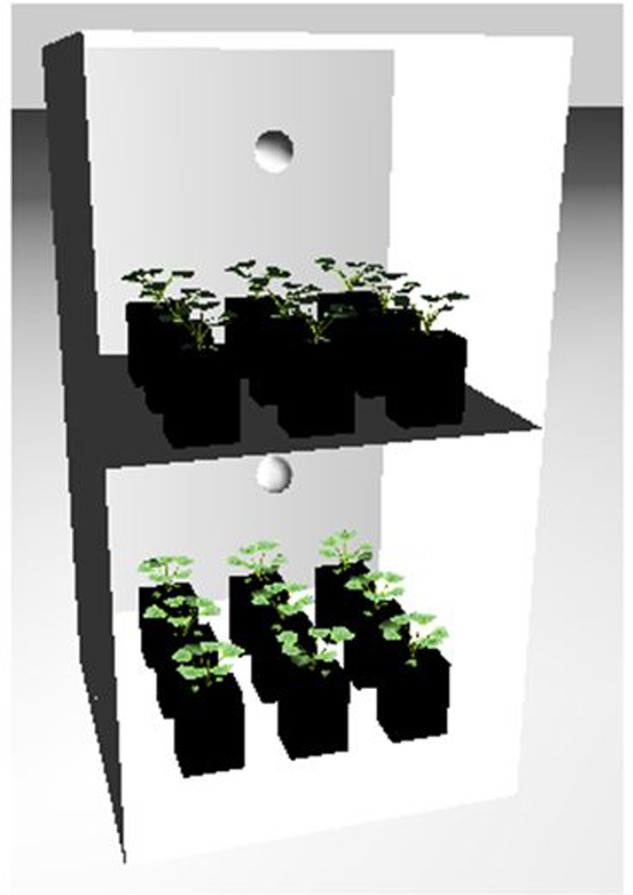
**Simulation of the temperature chamber as a 3D model in the Growth Grammar-related Interactive Modeling Platform (GroIMP) modeling environment, with area lights mapped onto the two side walls and the back wall, representing the 39 wall-mounted fluorescent light tubes (door removed for better visibility), and with 2 × 9 plants in pots on the two shelves.** For details, see Section “FSPM of Rapeseed Development.”

**FIGURE 6:**
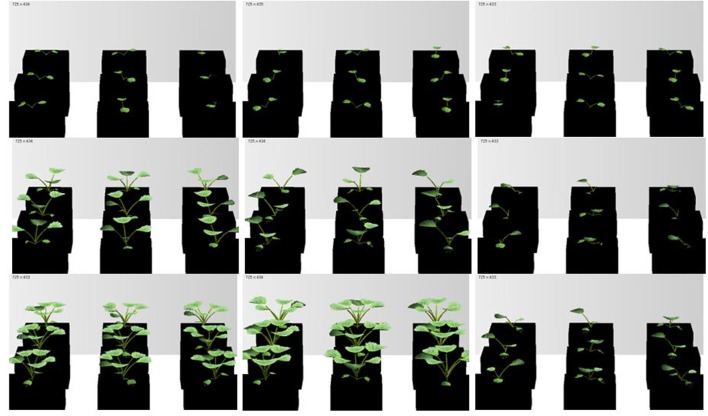
**Simulated rapeseed seedlings at three temperature treatments: *H*, *M*, and *L* temperature (left to right) and at different times after transfer (days after treatment, dat) to the chamber: 2, 13, and 20 dat (top to bottom)**.

**FIGURE 7:**
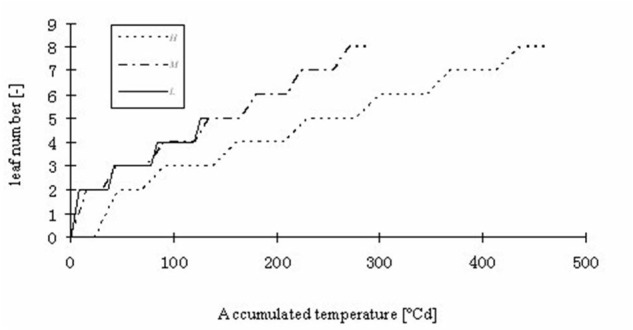
**Simulated leaf number kinetics of rapeseed seedlings at three temperature treatments: *H, M*, and *L* signify the plants under high, medium, and low temperature treatment, respectively**.

**FIGURE 8:**
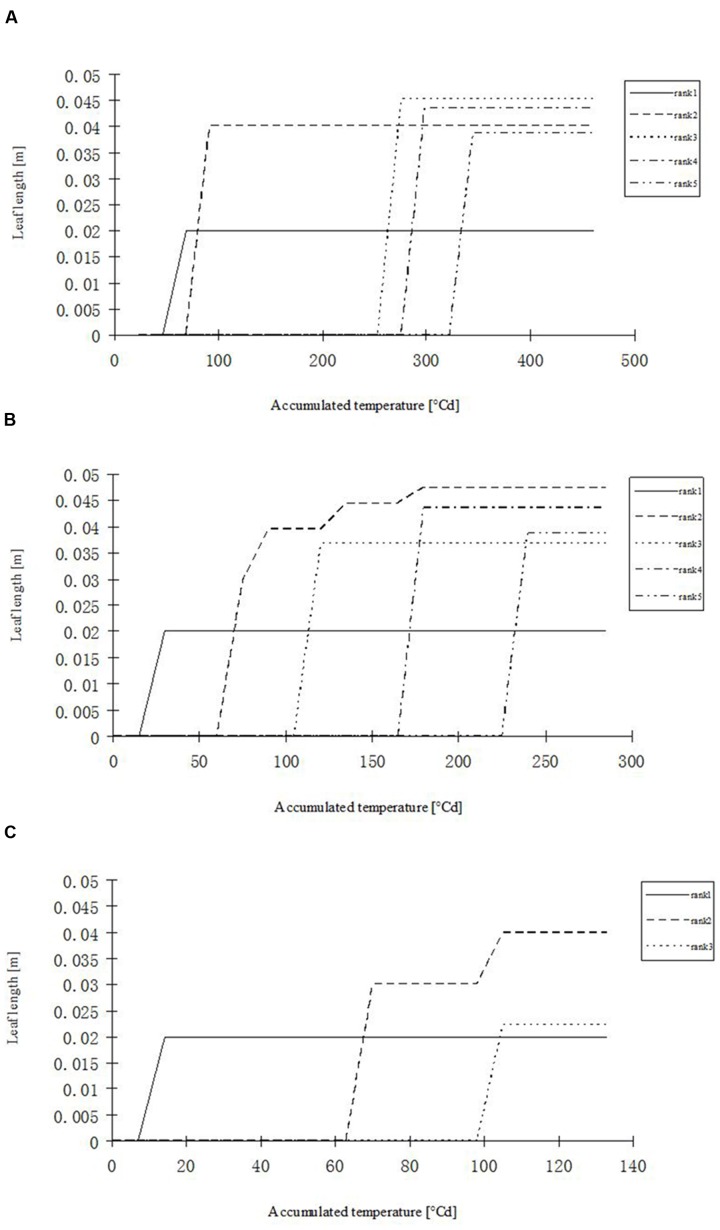
**Simulated leaf blade length development in rapeseed seedlings of different leaf ranks at three temperature treatments.** **(A)** *H* temperature treatment, **(B)** *M* temperature treatment, **(C)** *L* temperature treatment.

**FIGURE 9:**
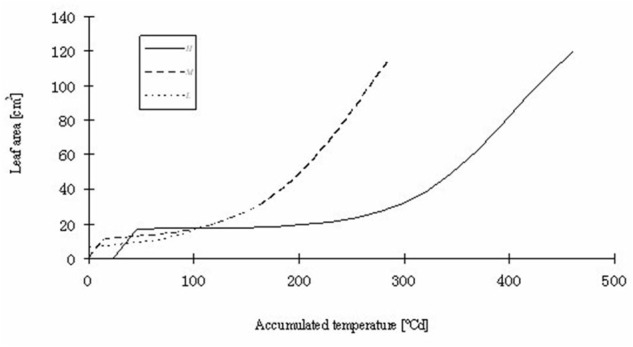
**Simulated total leaf blade area of rapeseed seedlings at three temperature treatments.** *H, M*, and *L* signify the plants under high, medium, and low temperature treatment, respectively.

**FIGURE 10:**
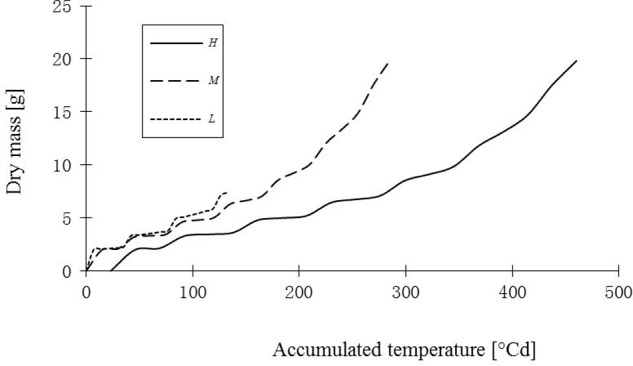
**Simulated total dry mass of leaves of rapeseed seedlings under three temperature treatments.** *H, M*, and *L* signify the plants under high, medium, and low temperature treatment, respectively.

Some basic parameters used in the rapeseed model are listed in **Tables 1**–**4**. We used leaf appearance rates derived from data as (**Figure 1**), describing the kinetics of leaf appearance, expressed as the number of visible leaves as a function of time (days after transfer into the climate chambers). The daily accumulated temperature increment, as derived from the slope, minus the temperature for growth of rapeseed (5°C) (Tang et al., 2007, 2009) was 23°Cd d^-1^, 15°Cd d^-1^, and 7°Cd d^-1^, at temperature regimes of 26/30°C, 18/22°C, and 10/14°C, respectively. Some derived morphological parameters were calculated, such as the width/length ratio (WLR), the petiole-blade ratio (PBR) and leaf mass per area (LMA) (**Figures 2**–**4**). Integration of these elements into the FSPM-P yielded a first model version in which the rough shape of the leaves could be represented (**Figures 5**, **6**). Finally, total dry mass, total leaf area of the whole plant, and leaf mass per area (LMA) of each leaf from our data were used to parameterize the growth curves, which in turn were used to simulate leaf extension (**Figures 7–9**) and dry matter accumulation kinetics (**Figure 10**).

## Results

The fitted model parameters for the first five leaf blade ranks and temperature treatments are given in **Table 1**. The first two true leaves (except cotyledons) of oilseed rape were usually small, and preformed in the seed. As the first leaf was already present or fully extended by the time of the start of the experiment, the fit of timing (*x*_0_) and slope (*b*) parameter of the logistic equation was not very good. A true sigmoid curve was only fitted for leaf ranks of three and above (**Figure 1**). Furthermore, at low temperature leaves were smaller and grew more slowly, also leaf appearance rate was slower, leading to fewer observed leaves within the observation period (**Figure 1**).

### Morphological Parameters

The relationship between width and length of the leaves at different temperatures was expressed as WLR. The WLR decreased with leaf rank. Young leaves had a tendency to be narrower than older ones (**Figure 2**). There was a clear relationship between PBR and leaf rank (**Figure 3**), changing in a linear fashion with rank. More specifically, PBR increased with increasing rank at the *H* treatment, whereas it decreased at the other two treatments.

### Total Dry Mass and Total Leaf Area of the Whole Plant

At the first sowing date, the differences in leaf area and leaf dry weight between the *H* and *M* treatment were not significant (*P* = 0.01; **Table 5**). Only the low temperature treatment was significantly different from the other two, by producing leaves with smaller area and lower dry mass (*P* = 0.01). However, for the second sowing date, all three treatments significantly differed from each other with respect to both parameters (*P* = 0.01; **Table 5**).

**Table 5:** Total leaf area and dry weight of the whole plant in *B. napus* seedlings under three temperature treatments.

Parameter	Sowing date	Temperature treatment		
		
		*H*	*M*	*L*
Leaf area [cm^2^]	First sowing date	23.63 a^∗^	25.31 a	9.36 b


	Second sowing date	68.27 b	85.41 a	18.16 c
Leaf dry weight [mg]	First sowing date	31.86 a	35.59 a	14.13 b


	Second sowing date	109.50 b	139.49 a	27.54 c


**H, M*, and *L*: high, medium, and low temperature treatments.*

*^∗^ Within each temperature treatment, means followed by the same letter are not significantly different according to the LSD test at *P* ≤ 0.01. ‘a’ is the highest and ‘c’ is the lowest.*

With respect to the duration of treatment (**Table 6**), it was observed that at the earlier sowing date a significant difference in leaf area occurred between treatment duration of 6 and 9 days, and 12 and 15 days, respectively. A similar pattern was observed for leaf dry weight, but additionally, dry weights differed already between 3 and 6 days duration (*P* = 0.01).

**Table 6:** Total leaf area and dry weight of the whole plant in *B. napus* seedlings at different temperature treatment durations.

First sowing date	Parameter	Treatment duration [d]						
		
		3	6	9	12	15		
	Leaf area [cm^2^]	13.03 c^∗^	12.21 c	20.28 b	19.46 b	32.17 a		
	Leaf dry weight [mg]	9.22 d	19.10 c	29.18 b	28.77 b	49.68 a		

**Second sowing date**		**3**	**6**	**9**	**12**	**15**	**18**	**21**

	Leaf area [cm^2^]	10.58 d	19.81 d	26.56 cd	47.69 c	82.67 b	101.22 ab	112.44 a
	Leaf dry weight [mg]	17.94 d	34.18 cd	46.1 cd	76.38 c	130.01 b	159.73 ab	180.92 a


*^∗^ Within each row, means followed by the same letters are not significantly different according to the LSD test at *P* ≤ 0.01. ‘a’ is the highest and ‘d’ is the lowest.*

### Leaf Mass Per Area (LMA) of Individual Leaves at Different Treatments

Generally, with the exception of the *L* treatment, LMA significantly increased with rank (**Figure 4**). The regression between leaf rank *r* and LMA was, for high temperature: *LMA* = 0.0133*e^0.0585r^* (*R*^2^ = 0.9585, *n* = 102), and for medium temperature: *LMA* = 0.0143*e^0.0607r^* (*R*^2^ = 0.8195, *n* = 108). This relationship was not significant for the low temperature treatment.

### Simulation Output

Part of the observations and data given above were used to set up and parameterize the functional–structural model in order to integrate the different results into a spatially explicit form, for the different temperature treatments. Simulations of some developmental stages of young rapeseed plants are shown in **Figures 5**, **6**. As the measured phyllochron was an input to the model, the accordance between measured and simulated leaf appearance rate naturally was very good (**Figure 7**). Simulated expansion dynamics of leaf blades of different ranks and at different temperatures are shown in **Figure 8**. Simulated blade expansion subsided more rapidly and abruptly than in reality (cf. **Figure 1**). The total simulated leaf area at different temperatures, based on the leaf area dynamics function (Equation 4) is shown in **Figure 9**. At the medium temperature treatment simulated leaf area increased faster than under low or high temperature treatment, respectively. Curves were similar among different temperature treatments. However, growth at the medium temperature treatment seemed to be faster than at the high temperature treatment.

## Discussion

Temperature is a major factor for plant growth rate and daily photosynthesis (Rapacz et al., 2001, 2003; Qaderi et al., 2006, 2010, 2012; Kromdijk and Long, 2016; Zhang et al., 2016). Furthermore, it has also an influence on oil quality (Baux et al., 2008, 2013). In the present study, the effect of temperature on leaf expansion was investigated in oilseed rape. Width-length ratio (WLR) at different temperatures exhibited significant differences only at high temperature (WLR = 0.849). In the *M* and *L* treatments PBR of the upper leaves was small, which means that the petiole was shorter relative to the blade. Overall, the decrease of PBR with rank will result in a conical leaf arrangement (with upper leaf blades being less distant from the main stem), which could be a plant-level strategy to increase light penetration to the lower leaves. More experimental work will be required to determine the exact reasons for the observed patterns. The PBR is one factor, besides WLR, determining leaf shape. Its change along the main stem can be interpreted in terms of developmental progress from the vegetative to the reproductive stage, with lower-ranked leaves being, in principle, more “vegetative”. The increase in total leaf area and dry mass was lowest under the low temperature treatment. In a study similar to the present one leaf area decreased while leaf blade thickness increased when rapeseed plants were exposed to cold temperatures (Stefanowska et al., 1999). Furthermore, there were no significant differences in leaf area between medium and high temperature at the beginning of juvenile stage. However, when treatment duration was long enough (>6 or >10 days, **Table 6**), leaf area and dry mass at medium temperature were increasing faster than at high temperature. Thus, it can be concluded tentatively that an accumulated temperature increment of 75°Cd is necessary as a phyllochron for the formation of a leaf on the main stem.

Leaf mass per area (LMA) was correlated with environmental and topological factors, such as temperature and leaf rank. LMA was positively correlated with temperature; similar research was also found in other studies (Gausman et al., 1971; Qaderi et al., 2006; Jullien et al., 2009). In the present study, the average LMA under the *H*, *M*, and *L* treatments were 0.01328 kg m^-2^, 0.01766 kg m^-2^, and 0.01639 kg m^-2^, respectively. There was a significant difference between the *H* treatment and the other two treatments. However, *M* and *L* did not differ significantly from each other. A more specific experimental study would be required to obtain a better idea concerning this phenomenon. LMA of upper leaves was higher than that of lower leaves, confirming the findings of Gausman et al. (1971). This effect was not significant for the three or four adjacent ranks in each treatment (**Figure 4**). The absence of a significant difference at the *L* treatment could be explained by the fact that only four leaves per main stem were formed in total.

Plant architecture is of major agronomic importance for the adaptability of a plant for cultivation, and to predict how the plant grows under different environments (Reinhardt and Kuhlemeier, 2002). The impact of plant architecture and morphology on the light climate within the plant canopy and the resulting amount of light locally incident, absorbed by a given assimilating surface, and finally used for photosynthesis plays a major role in plant development as it determines local assimilate availability and thus source strength (Evers et al., 2006). To investigate this complex context and the underlying relationships, we implemented a FSPM as an extension of the FSPM-P model devised by Henke et al. (2016). This model, apart from having a generic description of the sink behavior of young leaves and internodes, also contains provisions to simulate generically photosynthesis on a per-leaf basis, using local light and temperature as an input. Employing a prototype as a departure point permitted us to obtain a usable model relatively rapidly, as the only major steps to be done were parameterization and, eventually, the writing of simple extensions of rules to accommodate crop-specific processes that were not covered by the general model. The FSPM-P is written in the language XL which is included in the GroIMP platform (Kniemeyer et al., 2007; Kniemeyer, 2008), with specialized tools for plant modeling. GroIMP provides an adequate radiation model that can compute the amount of light absorbed by each plant organ (more generally, objects in a simulated scene; Hemmerling et al., 2008). For more advanced studies of the impact of light quality (e.g., red to far-red ratio) on growth processes, spectral light can be simulated (Henke et al., 2016). The new light model makes use of parallel processing on a graphical processing unit of the computer, thereby accelerating computation enormously (van Antwerpen et al., 2011).

In recent years, models of the main agricultural crops (de Carvalho Lopes and Steidle Neto, 2011) have been developed and their usefulness and importance has been increasingly recognized by the agronomical community (Vos et al., 2010). The most striking advantage of a modeling approach is the time that can be saved when employing a model to predict yield or quality features of a new crop, compared with traditional methods of experimentation and breeding in the field. At the same time, the vivid and realistic visualization of plant development in the case of a 3D model renders teaching much easier and more attractive (Fourcaud et al., 2008). Apart from that, virtual plants or FSPMs, usually integrate a lot of information about the morphology of the crop in question as well as about the nature and spatiotemporal dynamics of physical and biological processes (Buck-Sorlin, 2013). Thus, such models that are capable of integrating the dynamics of architectural development with the rate of photosynthesis and respiration are an excellent means to estimate the production of future biomass of a plant (Godin and Sinoquet, 2005).

In the field, a rapeseed plant stops growth below 5°C; whereas photosynthesis usually continues well below this threshold temperature for growth (Tang et al., 2007, 2009). This leads to products of photosynthesis accumulating in the plant (as starch), where the extent of accumulation will be a function of photosynthetically active leaf area as well as of the accumulated temperature favorable for photosynthesis (below the threshold for growth yet above the threshold for photosynthesis and above that leading to frost damage). Consequently, regrowth of the plant in spring, as temperatures rise again to favorable values for growth, will be the more rapid the more reserves the plant has been able to accumulate during winter. In a future version of the model, we will link the rate of regrowth of rapeseed in spring with the amount of stored starch during the winter (Martre et al., 2011). In doing so, an existing photosynthesis module [based on the LEAFC3 model by Nikolov et al. (1995)] will be reparameterized from the literature (Leng et al., 2002; Liu et al., 2003; Ma et al., 2009) in order to obtain more accurate values for parameters influencing the rates of photosynthesis and respiration of the young rapeseed. It is possible to compute the total dry mass from an estimate of the amount of carbon fixed by photosynthesis and the amount of carbon lost through maintenance and growth respiration.

The primary aim of the present study was to build a model of the young stages of rapeseed in the leading commercial cultivar (*Brassica napus* L. cv. ZS 758). In order to build a robust and representative model in the future we will need to consider an experimental setup consisting of more plants and several, contrasting rapeseed cultivars. In addition, as a further step, the current model will be extended to also represent further development from regrowth in spring to maturity (early summer). If successfully parameterized and calibrated such a model could be used to predict harvestable yield, as a function of architectural development at the seedling and juvenile stages. This could in consequence allow the use of such a model as a tool to optimize yield by proposing leaf architecture ideotypes optimized for a given temperature regime in autumn and winter, respectively, an optimal climate dynamics for a given cultivar.

## Conclusion

The present study yielded new knowledge about the influence of temperature on leaf expansion in the early development of oilseed rape by combination of observed and literature-derived data. This combination is feasible and useful, as a complete measured dataset is almost never available. Furthermore, embedding of the datasets into an FSPM delivered interesting insights into the interactions of organogenetic and morphogenetic processes with temperature and into source–sink interactions in particular.

## Author Contributions

GB-S and WZ conceived the study. TT, LW, MH, and BA conducted experiments and analyzed data. MH and GB-S adapted the model. TT, MH, GB-S, and WZ wrote and revised the manuscript. All authors read and approved the manuscript.

## Conflict of Interest Statement

The authors declare that the research was conducted in the absence of any commercial or financial relationships that could be construed as a potential conflict of interest.

## Footnotes

1 http://www.tutiempo.net/en/Climate/Hangzhou/584570.htm
